# Effects of duodenal switch alone or in combination with sleeve gastrectomy on body weight and lipid metabolism in rats

**DOI:** 10.1038/nutd.2014.22

**Published:** 2014-06-30

**Authors:** O A Gudbrandsen, Y Kodama, S A Mjøs, C-M Zhao, H Johannessen, H-R Brattbakk, C Haugen, B Kulseng, G Mellgren, D Chen

**Affiliations:** 1 Department of Clinical Medicine, University of Bergen, Bergen, Norway; 2 Department of Clinical Science, University of Bergen, Bergen, Norway; 3 Department of Cancer Research and Molecular Medicine, Norwegian University of Science and Technology, Trondheim, Norway; 4 Department of Chemistry, University of Bergen, Bergen, Norway; 5 Nofima BioLab, Fyllingsdalen, Norway; 6 Hormone Laboratory, Haukeland University Hospital, Bergen, Norway; 7 KG Jebsen Center for Diabetes Research, University of Bergen, Bergen, Norway; 8 Department of Surgery, St Olav's University Hospital, Trondheim, Norway

## Abstract

**Background:**

A combined procedure of sleeve gastrectomy and duodenal switch (SG+DS) has been applied to the treatment of super obesity. The aim of the present study was to test whether duodenal switch alone (DS) leads to similar weight loss and changes in lipid metabolism as SG+DS.

**Methods:**

Male Sprague–Dawley rats underwent sham surgery (Sham, *N*=7), duodenal switch alone (DS, *N*=5) or sleeve gastrectomy followed by duodenal switch (SG+DS, *N*=5). Body weight, feed and water intakes, and ambulatory activity were recorded 2 months post surgery. Tissue and faecal lipids, faecal bile acids, plasma cytokines and lipid metabolism-related gene expression in adipose tissue and liver were analysed.

**Results:**

Daily energy intake, relative feed uptake, ambulatory activity and body weight reduction were similar between DS and SG+DS rats. The hepatic triacylglycerol content was higher and faecal secretion of triacylglycerol was lower after SG+DS compared to DS (*P*<0.05). Faecal bile acid secretion was higher in SG+DS than in DS rats (*P*<0.05) despite similar hepatic CYP7A1mRNA level. Plasma levels of proinflammatory cytokines interleukin (IL)-1b, IL-2, IL-4, IL-5, IL-6, IL-12, granulocyte-macrophage colony stimulating factor and tumour necrosis factor alpha were higher in SG+DS than in DS rats (*P*<0.05).

**Conclusions:**

Although DS and SG+DS had similar efficacy in terms of body weight loss, SG+DS resulted in a poorer regulation of lipid metabolism than DS.

## Introduction

The prevalence of obesity has reached epidemic proportions and is one of the leading public health concerns on a worldwide scale.^1^ Obesity causes or exacerbates many health problems, and is associated with the development of diseases such as coronary heart disease, mediated through disturbances of lipid metabolism and blood pressure.^2^

Strategies for weight loss include reduced energy intake from foods, increased physical activity, medical treatment and bariatric surgery. For individuals with severe obesity (body mass index >40 kg m^−2^), dietary therapy, even together with exercise and behaviour therapy, is rarely successful,^3^ and most medical or behavioural interventions result in only 5–10 percentage excess weight loss.^4^ The most effective and recognized method for treating morbid obesity is bariatric surgery.^5, 6, 7^

Bariatric surgery is believed to reduce the nutrient uptake, either by restricting the energy intake by procedures such as sleeve gastrectomy (SG) that reduces the volume of the stomach and thus the amount of food eaten, or by reducing the uptake from the intestinal tract after bypassing parts of the small intestine by procedures such as duodenal switch (DS), or a combination of restrictive and malabsorptive elements such as sleeve gastrectomy and duodenal switch (SG+DS) or gastric bypass (GB). Very recently, this dogma has been challenged by an animal study in which SG surgery was applied to mice.^8^ The study points to bile acids and farsenoid-X receptor signalling as an important molecular underpinning for the beneficial effects of this weight-loss surgery.

GB is the most common procedure because of its relatively high efficacy and safety, whereas the combination of SG and DS (SG+DS) has been shown to be even more effective than GB, particularly in super-obese patients in terms of body weight loss^9^ as well as improvement of comorbidities such as diabetes, hypertension and dyslipidemia.^10, 11, 12^ Recently, we have reported that GB induced body weight loss mainly by increasing energy expenditure, whereas SG+DS induced greater body weight loss by reducing food intake, increasing energy expenditure and causing malabsorption in rat models.^13^

Previously, we have shown that DS without SG could be an independent bariatric procedure for inducing weight loss in rats.^14^ Later, a study showed that SG (without DS) led to a marked, weight-independent reduction in secretion of intestinal triglycerides, and suggested that SG has important effects on lipid metabolism.^15^ Thus, in the present study we wanted to test our hypothesis that the combination of SG+DS exhibits beneficial effects not only in body weight loss but also in lipid metabolism by using rat models of surgery.

## Materials and Methods

### Animals and diets

Male Sprague–Dawley rats (Taconic M&B, Skensved, Denmark) aged 3 months were housed at a 12-h light–dark cycle, 22°C and 40–60% humidity. All rats had free access to tap water and feed (RM1, Special Diets Services, Witham, UK).

### Experimental design

Rats aged 3 months were divided into three experimental groups: duodenal switch alone (DS, *N*=7), sleeve gastrectomy followed by duodenal switch (SG+DS, *N*=6) and sham operation (controls, *N*=7). Sample sizes were kept small in consideration of the ‘3Rs' for the humane use of animals (for example, reduction of animal numbers to the minimum consistent with achieving the scientific purposes of the experiment).^16^

DS, SG and sham surgery were performed when the rats' average body weight was 525 g, and DS following SG was performed 3 months after SG. The numbers of surviving rats in the experimental groups were 5 DS rats, 5 SG+DS rats and 7 sham rats. The rats were euthanized 2 months after their last surgical procedure, while they were under anaesthesia with 2–4% Isofluran (Baxter Medical AB, Kista, Sweden) mixed with nitrous oxide and oxygen. It cannot be excluded that the difference in age at the time of sacrifice may make it difficult to dissociate procedure from ageing effects. However, based on our previous study of 1-year follow-up after bariatric surgery (for example, GB), there would unlikely be big differences within a window of 3–6 months.^17^

Blood was drawn from the abdominal aorta and EDTA added. Liver, skeletal muscle from the thigh and epididymal white adipose tissue were instantly removed and frozen in liquid nitrogen.

The experimental protocol was approved by the Norwegian Animal Research Authority.

### Surgery

Surgeries were performed under general anaesthesia (Isofluran, Baxter Medical AB). SG was performed by resecting 75% of the glandular stomach along the greater curvature. DS was constructed by transecting the duodenum 1 cm to the pylorus, and a common channel was created by dividing the ileum 5 cm proximal to the ileocaecal junction. The distal limb of the ileum was anastomosed to the post-pyloric duodenum in an end-to-end manner, and the stump of the duodenum was closed with cross-suture. The distal anastomosis was performed by joining the distal biliopancreatic limb at 1 cm to the ileocaecal junction in an end-to-side manner.

### Eating and metabolic parameters

Rats were placed in the cages for a comprehensive laboratory animal monitoring system (CLAMS; Columbus Instruments International, Columbus, OH, USA) for registration of feed and water intake, registration of ambulatory activity and collection of faeces for 48 h with free access to standard rat powder feed (RM1) and tap water.

### Lipids in tissues and faeces

Lipids were extracted from the liver, skeletal muscle and faeces by the method of Bligh and Dyer,^18^ evaporated under nitrogen and re-dissolved in isopropanol before analysis on Cobas c111 system (Roche Diagnostics GmbH, Mannheim, Germany) using the appropriate kits from Roche Diagnostics GmbH.

### Total faecal bile acids

Faecal total bile acids (3α-hydroxy bile acids) were measured in freeze-dried faeces,^19^ using Chromabond C18ec (3 ml/200 mg, Macherey-Nagel, Düren, Germany) and the enzymatic bile acid kit from Sigma Diagnostics (St Louis, MO, USA) on the Cobas c111 system.

### Fatty acids in liver and skeletal muscle

Lipids were extracted from liver and muscle samples using a mixture of chloroform and methanol.^18^ Heneicosanoic acid was added to the extracts as internal standard. The extracts were methylated as described previously^20^ and extracted twice with isooctane. The methyl esters were quantified by an 7890 gas chromatograph (Agilent, Santa-Clara, CA, USA) equipped with a flame ionization detector and a BPX-70 capillary column as described in Sciotto and Mjos^21^ with minor adjustments of the temperature programme. The compounds were identified by gas chromatography–mass spectrometry using the BPX-70 column and methodology as described in Wasta and Mjos.^22^

### qRT-PCR

Total RNA was purified from epididymal white adipose tissue and liver using the RNeasy Lipid Tissue Midi Kit for adipose tissue and the RNeasy Mini Kit for liver samples (Qiagen, Hilden, Germany). qRT-PCR was carried out in triplicate using the LightCycler480 instrument, Probes Master kit and the LightCycler480 rapid thermal cycler system (Roche Diagnostics GmbH), and complementary DNA was amplified using target-specific primers and Universal ProbeLibrary (UPL) probes (Roche Diagnostics GmbH). The following primers and UPL probes were used: low-density lipoprotein receptor (LDLr, UPL #16), acetyl-CoA carboxylase (Acaca, UPL #63), sterol *O*-acyltransferase (Soat2, UPL #75), cholesterol 7 alpha-hydroxylase (Cyp7a1, UPL #110), HMG-CoA reductase (Hmgcr, UPL #122), glucose transporter GLUT2 (Slc2a2, UPL #122), glucose transporter GLUT4 (Slc2a4, UPL #67), 18S rRNA (Mrps18a, UPL #117), acidic ribosomal phosphoprotein P0 (Arbp, UPL #85) and ribosomal protein L32 (Rpl32, UPL #117). Of the three reference genes tested (Arbp, Mrps18a and Rpl32), Mrps18 was least affected by surgical interventions; therefore results are shown relative to Mrps18a and normalized to controls.

### Plasma levels of cytokines

Cytokines in plasma were analyzed using a multiplex cytokine assay (Cat no.: 171-K1002M, Bio-plex Pro Rat Cytokine Th1/Th2 12-plex Panel; Bio-Rad Laboratories, Hercules, CA, USA) for interleukin (IL)-1a, IL-1b, IL-2, IL-4, IL-5, IL-6, IL-10, IL-12, IL-13, granulocyte-macrophage colony stimulating factor, interferon gamma and tumour necrosis factor alpha. The analysis was performed on a Luminex 100/200TM System (Luminex Corp., Austin, TX, USA)

### Statistical analysis

One-way ANOVA followed by a Tukey *post hoc* test was applied where appropriate for comparison between the experimental groups (PASW 19.0 software, IBM Corporation, Armonk, NY, USA). Principal component analysis and soft independent modelling of class analogies of the fatty acid results were performed using Unscrambler 9.8 (CAMO, Oslo, Norway). Prior to extraction of the principal components the data were normalized so that the fatty acids for each object summarized to 100%, and variables were divided by their own standard deviation (standardization) before the mean values were subtracted (centering). *P* values <0.05 were considered statistically significant.

## Results

### Bodyweight, growth, energy and water intake

The body weight was similar between all experimental groups at the time of the first surgery (Table 1). Twelve weeks after sleeve gastrectomy, the rats in the SG+DS group underwent duodenal switch, and no significant change was seen in body weight at the time of the second surgery. The body weight was similar between the DS and SG+DS groups at the time of euthanasia, and for both groups the average body weight was significantly lower compared to sham-operated rats (*P*=3 × 10^−7^ and 7 × 10^−7^, respectively). Concomitantly, the weight loss after surgery was similar between DS and SG+DS groups. The daily energy intake was similar between DS and SG+DS, whereas the water intake was lower in SG+DS rats than in DS rats (*P*=0.03). Both the daily energy and water intake were significantly lower in DS and SG+DS rats when compared to sham rats (*P*<0.001). The energy intake relative to body weight was similar between DS and SG+DS, but was significantly higher in DS when compared to sham rats (*P*=0.01). The water intake relative to body weight was significantly lower in SG+DS when compared to DS rats (*P*=0.006). The ambulatory activity was similar between all groups, both considering total counts per day and counts relative to energy intake.

### Levels of triacylglycerol and cholesterol in the liver and skeletal muscle

The hepatic triacylglycerol content was significantly higher (*P*=0.002) and the hepatic cholesterol content tended to be higher (*P*=0.068) in SG+DS when compared to DS (Table 2). The liver samples collected were frozen directly and were not chemically fixated for histological use, but Sudan black staining supported a higher lipid content in SG+DS rats (data not shown).

In muscle, no differences were seen in triacylglycerol and cholesterol content between DS and SG+DS. When compared to Sham rats, the triacylglycerol content was significantly lower and the cholesterol content was significantly higher in muscle after SG+DS (*P*=0.02 for both comparisons).

### Fatty acid composition in liver and muscle

**Figure 1:**
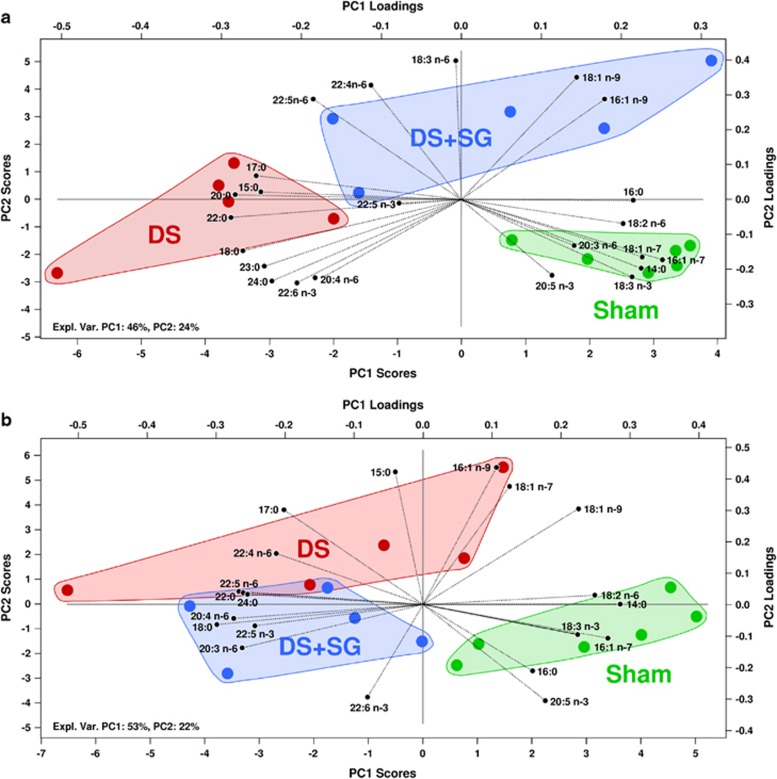
Principal component scores and loadings of fatty acid profiles in liver (**a**) and muscle (**b**).

The principal component analysis bi-plots of the fatty acid compositions in Figure 1 give an overview of the group differences in liver and muscle. In both liver and muscle the objects are separated into three distinct classes based on differences in fatty acid composition, but there is limited separation between DS and SG+DS. In liver (Figure 1a) DS is separated from the sham group along the first principal component (PC1), which explains 46% of the variance in the data, while DS+SG is separated from the sham group basically along PC2, which explains 24%. Thus, DS is more different from sham-operated rats. The plot of the muscle fatty acids (Figure 1b) shows the same trend as seen in liver, as sham rats are separated from DS and SG+DS rats basically by PC1 (53%) while the two operated groups are separated by PC2 (22%). Some trends in the fatty acid composition were seen in both liver and muscle, for instance that C18:2n-6, C14:0, C18:3n-3, C16:1n-7, C16:0 and C20:5n-3 were in the direction of the sham group. In both tissues, the long-chain n-6 PUFAs C22:4n-6 and C22:5n-6 were positioned in opposite directions from the sham group, indicating lower amounts in this group. The clearest effect of the surgeries on a single fatty acid was seen on C22:5n-6, which was much lower in all sham rats. Analysis by the soft independent modelling of class analogies showed that all DS and SG+DS rats were significantly different from sham at a 5% level in both liver and muscle.

The hepatic levels of total saturated fatty acids (SFA), C14:0, C15:0 and C17:0 were similar between DS and SG+DS rats, whereas the level of C16:0 was higher and that of the longer SFAs, C18:0, C20:0, C22:0, C23:0 and C24:0, were lower in SG+DS when compared to DS (*P*<0.05, Table 3). The total MUFA amount in liver was significantly higher in SG+DS when compared to DS (*P*=0.03), mainly due to higher level of C18:1n-9 in the SG+DS rats (*P*=0.02). The hepatic Σn-3 PUFA level was significantly lower in SG+DS when compared to DS rats (*P*=0.04), due to a high level of C22:6n-3 in SG+DS rats (*P*=0.01). In SG+DS rats the hepatic levels of C18:3n-6 and C22:4n-6 were higher and that of C20:4n-6 was lower when compared to DS (*P*<0.05), with no differences in total n-6 PUFA level between these groups. The ratio of C18:1n-9 to C18:0 was significantly higher in SG+DS compared to DS (*P*=0.03), implying that the activity of stearoyl-CoA desaturase was higher in the liver from SG+DS rats. The ratio of C20:4n-6 to C18:2n-6 was lower in the liver from SG+DS rats when compared to DS (*P*=0.04), implying a lower activity in delta-6 and/or delta-5 desaturases in SG+DS rats.

In skeletal muscle, the amounts of C15:0 and C17:0 were significantly lower in SG+DS compared to DS (*P*<0.05), and no differences were seen between these groups regarding total SFA (Table 3). Also, ΣMUFA was similar between muscle from DS and SG+DS, albeit the amount of C18:1n-7 was significantly higher in GS+DS rats (*P*=0.008). The total amount of n-3 PUFA and the level of the individual n-3 PUFAs were similar between DS and SG+DS rats. The total n-6 PUFA was significantly higher in GS+DS rats when compared to DS (*P*=0.001), although the amount of C18:2n-6 was significantly lower in the GS+DS rats (*P*=0.02). The ratios C18:1n-9/C18:0 and C20:4n-6/C18:2n-6 were similar between muscles from DS and SG+DS rats.

### Faecal output

No significant differences was seen in the total faecal mass or the dry faecal mass between the experimental groups (Table 2), but there was a tendency for a higher faecal mass in DS when compared to SG+DS (*P*=0.054). The relative feed uptake (moisture free feed intake/dry faecal material output) was similar between DS and SG+DS rats, and was significantly lower in both DS and SG+DS rats when compared to sham rats (*P*=1 × 10^−5^ and 8 × 10^−5^, respectively). The daily faecal triacylglycerol output was significantly higher in DS when compared to SG+DS (*P*=0.02), and tended to be higher when compared to sham rats (*P*=0.052). No significant differences were seen between the experimental groups regarding daily faecal cholesterol output; however, there was a tendency for a higher output in DS when compared to SG+DS (*P*=0.062). The daily faecal output of total bile acids was significantly lower in DS when compared to SG+DS and sham rats (*P*=0.03 for both comparisons).

### Expression of genes in WAT and liver

The mRNA level of acetyl-CoA carboxylase-α (Acaca) in white adipose tissue was similar between DS and SG+DS rats, and was significantly lower in DS (*P*=0.003) and tended to be lower in SG+DS (*P*=0.083) when compared to sham rats (Table 4), suggesting that the endogenous fatty acid synthesis could be lower in fat tissue after bariatric surgery, especially after DS. Both DS and SG+DS groups had similar mRNA levels of glucose transporter-4 (Slc2a4) and of LDL receptor (LDLr) in WAT, and both groups had lower mRNA levels of Slc2a4 (*P*=0.0003 and 0.003, respectively) and LDLr (*P*=0.03 and 0.04, respectively) when compared to Sham rats, implying a lower uptake of both glucose and LDL in adipose tissue after bariatric surgery.

No differences were seen between the groups regarding hepatic mRNA level of Acaca (Table 4). The hepatic mRNA levels of HMG-CoA reductase and Soat2, controlling cholesterol biosynthesis and the esterification of cholesterol, respectively, were similar between DS and SG+DS. The cholesterol metabolism in liver seems to be affected by bariatric surgery, as hepatic mRNA level HMG-CoA reductase was higher in SG+DS rats (*P*=0.04), and that of Soat2 was higher in both DS and SG+DS rats when compared to sham rats (*P*=0.0008 and 0.01, respectively). However, neither CYP7A1 nor LDLr hepatic mRNA levels, catalysing the production of bile acids from cholesterol and the uptake of cholesterol from circulation, respectively, were different between the DS, SG+DS and Sham rats. The mRNA level of the glucose transporter-2 gene (Slc2a2) was similar between DS and SG+DS rats, and was significantly lower in both DS and SG+DS groups when compared to sham rats (*P*=0.01 and 0.02, respectively).

### Plasma cytokines

The plasma levels of the cytokines IL-1a, IL-2, IL-4 and IL-5 were significantly higher in SG+DS rats when compared to DS rats (*P=*0.04, 0.005, 0.04 and 0.03, respectively), whereas no differences were seen between the groups regarding IL-1b, IL-10 and interferon gamma (Table 5). The plasma levels of IL-6, IL-12 p70, IL-13, granulocyte-macrophage colony stimulating factor and tumour necrosis factor alpha were below the limit of detection for DS rats only, and the plasma levels of these cytokines were similar between SG+DS and sham groups.

## Discussion

The surgical procedure of SG+DS is one of the best choices for inducing weight loss in super-obese patients. SG is getting increasingly more popular as the sole procedure for weight loss due to its efficiency of reducing excess weight, simplicity and the low occurrence of complications such as staple line leak.^23, 24, 25, 26, 27^

The DS procedure was originally created as a surgical solution for primary bile reflux gastritis and/or to decrease postoperative symptoms after distal gastrectomy and gastroduodenostomy.^28^ In patients, the operation usually consists of a 75% longitudinal gastrectomy (the so-called sleeve gastrectomy), creation of an alimentary limb that is ∼50% of the total small bowel length (that is, bypassing jejunum), a common channel length of 100 cm and cholecystectomy.^29^ In the present study using rats, DS was performed according to the rat anatomy. For instance, the rat stomach consists of antrum, fundus (also called corpus) and rumen (forestomach), while the human stomach is divided into antrum, body and fundus. The rat jejunum represents almost 90% of the small intestine, the human jejunum about 40%.^30^ Unlike humans, rats are nonemetic (not vomiting) and have no gallbladder. We show that in rats a similar weight loss was achieved in DS and SG+DS rats independently of the SG procedure as DS seems to be a determinant for the outcome in body weight. Concomitant with the similar body weight reduction, the energy intake and the ambulatory activity were similar between DS and SG+DS rats, although lipid metabolism seems to be differently affected after these bariatric procedures.

It was expected that DS should lead to a malabsorption whereas the combination of SG and DS should both reduce energy intake and lead to malabsorption. In the present study, the daily energy intake was similar between DS and SG+DS rats, and it was significantly lower compared to sham-operated rats. The water intake was also significantly lower in DS and SG+DS rats when compared to sham-operated rats. Although the faecal total output and the faecal dry mass were similar between all groups, the low relative feed uptake (feed intake/faecal output) in DS and SG+DS rats suggests that both surgical procedures lead to malabsorption.

The storage and faecal secretion of lipids seem to be differently regulated in DS and SG+DS rats, which is of interest as others have shown that hepatic lipid storage is not affected by SG alone.^15^ Whereas no significant difference was seen in liver triacylglycerol content between DS and sham rats, the hepatic triacylglycerol level in SG+DS was markedly higher compared to both groups, suggesting a poorer regulation of lipid metabolism after SG+DS. No differences were seen in muscle triacylglycerol content between DS and SG+DS. Also, the faecal output of triacylglycerol was significantly higher and the faecal bile acid secretion was significantly lower in DS when compared to SG+DS, possibly explaining the reduced storage of triacylglycerol in liver from DS rats when compared to SG+DS.

No differences were seen between DS and SG+DS for any of the genes investigated in liver. The higher mRNA expressions of HMG-CoA reductase and Soat2 in SG+DS livers compared to sham-operated rats suggest an increased production of cholesterol and cholesteryl esters for storage, but no difference was seen in hepatic total cholesterol between the experimental groups. Concomitant with this, the mRNA expression of LDL receptor and CYP7A1 in liver was similar between all groups. In addition, the mRNA expression of LDL receptor in WAT was significantly lower in SG+DS rats compared to sham-operated rats, suggesting that the cholesterol uptake in WAT was markedly lower in SG+DS rats. In SG+DS rats, the storage of cholesterol in muscle was similar to DS but significantly higher when compared to sham-operated rats. The faecal excretion of both cholesterol and bile acids in SG+DS rats was similar to sham-operated rats, thus suggesting that there is a change towards enhanced storage of cholesterol in skeletal muscle after SG+DS in rats. This further supports the notion that the lipid metabolism is poorer after SG+DS than after DS alone.

The mRNA expression of Acaca in WAT was similar between DS and SG+DS. The lower Acaca WAT mRNA level in DS rats compared to sham-operated rats suggests that the fatty acid synthesis was lower in DS rats; however, the expression of Acaca in WAT was similar between SG+DS and sham-operated rats. In liver, the Acaca mRNA level was similar between all groups, suggesting that the lower hepatic triacylglycerol level in DS compared to SG+DS rats was not controlled through Acaca. The high hepatic level of C18:1n-9 in SG+DS relative to DS corresponds well with the higher triacylglycerol content in SG+DS, and the high ratio of C18:1n-9 to C18:0 suggests that stearoyl-CoA desaturase was upregulated to promote increased storage of neutral fat in the liver of SG+DS rats.

The low mRNA level of Slc2a4 (GLUT4) in WAT in DS and SG+DS rats suggests that the capacity for uptake of glucose was reduced in these rats after surgery. A similar trend was seen in liver, with a significantly lower mRNA level of Slc2a2 (GLUT2) in DS and SG+DS rats, which is in line with reports of lower hepatic Slc2a2 mRNA level in diabetic rats after DS.^31^ A decrease in the uptake of glucose to WAT and liver may suggest a reduced conversion of glucose to triacylglycerol for storage in these tissues after DS alone or the combined SG+DS.

The principal component analysis of fatty acids in liver and muscle reveals that DS rats were not positioned between SG+DS and sham-operated rats as could be expected, indicating that there are different mechanisms that lead to the separation based on differences in fatty acid composition. The fatty acids in the direction of the DS and DS+SG rats are minor fatty acids, except C18:0, C20:4n-6 and C22:6n-3, which are typically found in membrane lipids.^32^ This observation may therefore reflect a reduced proportion of other lipid classes in these groups relative to the control. In liver, the ratio of C20:4n-6 to C18:2n-6 was significantly higher in DS compared to SG+DS, suggesting higher activities of delta-5 and delta-6 desaturases in DS. Concomitant with this, the triacylglycerol content was significantly lower in DS. The finding of possible lower delta-5 and delta-6 desaturase activities and higher amounts of triacylglycerol in the liver from SG+DS rats is in line with findings by others that the activities of these desaturases are low in obese non-alcoholic fatty liver patients.^33^

To see whether the poor lipid metabolism after SG+DS was accompanied with adverse changes in inflammatory markers, a panel of cytokines was analysed in plasma samples. Based on our results, it seems that the inflammatory status was worse after SG+DS than after DS alone, since the plasma levels of the proinflammatory cytokines IL-1a, IL-2, IL-4, IL-5, IL-6, IL-12, granulocyte-macrophage colony stimulating factor and tumour necrosis factor alpha were higher in the SG+DS group than in the DS group. Thus, our results show that the adverse effects on lipid metabolism after SG+DS compared to DS alone are accompanied with a worsening of the inflammatory status in SG+DS rats.

Taken together, our results suggest that DS (without additional SG) may be considered as a sole surgical procedure for obesity treatment. Although SG followed by DS led to similar weight loss, the effects by the two procedures on lipid metabolism inflammatory markers were different. Compared to DS, SG+DS rats had a reduced faecal output of triacylglycerol and an increased accumulation of triacylglycerol in liver that might lead to liver damage over time. Further investigations of lipid metabolism and the possible liver effects after SG+DS in patients should be considered.

## Figures and Tables

**Table 1:** Bodyweight at times of surgeries and euthanasia, growth, daily energy and water intake, and ambulatory activity at 8 weeks after surgery

	*Sham (*N=*7)*	*Duodenal switch (*N=*5)*	*Sleeve gastrectomy+duodenal switch (*N=*5)*
Body weight at time of first surgery, g	570±70	529±40	549±66
Body weight at time of second surgery, g	No surgery	No surgery	537±44
Body weight at time of euthanasia, g	589±77^a^	257±39^b^	276±37^b^
Weight change, percent	3±5^a^	−52±5^b^	−48±7^b^
Energy intake,^1^ kJ per 24 h	217±33^a^	132±13^b^	117±25^b^
Energy intake, kJ per kg BW	356±66^a^	496±44^b^	407±90^a,b^
Water intake, ml per 24 h	14.3±2.1^a^	7.9±2.9^b^	4.1±1.4^c^
Water intake, ml per kg BW	23±4^a,b^	30±10^a^	14±6^b^
Ambulatory activity, counts per 24 h	5784±1808	2934±1844	4133±2075
Ambulatory activity, counts per kJ energy intake	28±11	22±14	33±14

Results are presented as means±s.d. Means between groups were tested with one-way ANOVA using Tukey *post hoc* test. Means in a row with different letters are significantly different (*P*<0.05).

1 Digestable energy 11.90 MJ per kg feed (2.47% digestible oils, 12.92% digestible protein and 4.05% sugar).

**Table 2:** Levels of triacylglycerol and cholesterol in liver and skeletal muscle, and faecal mass, relative feed uptake and faecal secretion of triacylglycerol, cholesterol and total bile acids in faeces

	*Sham (*N=*6)*	*Duodenal switch (*N=*5)*	*Sleeve gastrectomy+duodenal switch (*N=*5)*
Liver triacylglycerols, μmol per g liver	12.1±3.7^a,b^	4.4±2.4^a^	19.2±8.2^b^
Liver total cholesterol, μmol per g liver	5.6±0.6	5.0±0.5	6.1±0.9
Muscle triacylglycerols, μmol per g muscle	7.6±3.9^a^	3.7±2.6^a,b^	2.2±1.1^b^
Muscle total cholesterol, μmol per g muscle	1.6±0.2^a^	2.5±0.7^a,b^	2.9±1.1^b^
Faeces, g per 24 h	4.6±1.1	5.9±0.7	4.1±1.4
Faeces, g dry material per 24 h	3.3±0.7	4.1±0.5	3.1±0.9
Relative feed uptake (dry feed/dry faecal weight)^1^	5.1±0.7^a^	2.4±0.3^b^	2.9±0.7^b^
Faecal triacylglycerol, μmol per 24 h	6.2±3.4^a,b^	13.5±6.9^a^	4.3±4.0^b^
Faecal total cholesterol, μmol per 24 h	18.8±7.2	25.9±9.8	14.0±5.2
Faecal total bile acids, μmol per 24 h	34.0±16.5^a^	12.3±7.6^b^	35.9±10.8^a^

Results are presented as means±s.d. Means between groups were tested with one-way ANOVA using Tukey *post hoc* test. Means in a row with different letters are significantly different (*P*<0.05).

1 Moisture content in feed was 10%.

**Table 3:** Fatty acid compositions in liver and skeletal muscle

*g per 100 g FA*	*Liver*			*Skeletal muscle*		
	*Sham (*N=*6)*	*Duodenal switch (*N=*5)*	*Sleeve gastrectomy+duodenal switch (*N=*5)*	*Sham (*N=*6)*	*Duodenal switch (*N=*5)*	*Sleeve gastrectomy+duodenal switch (*N=*5)*
C14:0	0.54±0.08^a^	0.31±0.09^b^	0.40±0.08^b^	0.92±0.33^a^	0.46±0.21^b^	0.41±0.17^b^
C15:0	0.22±0.01^a^	0.37±0.05^b^	0.28±0.12^a,b^	0.16±0.02^a^	0.23±0.05^b^	0.15±0.03^a^
C16:0	22.99±0.89^a^	19.81±0.87^b^	22.98±1.24^a^	24.40±1.63	21.76±2.13	24.00±1.32
C17:0	0.29±0.03^a^	0.69±0.15^b^	0.54±0.26^a,b^	0.26±0.04^a^	0.53±0.12^b^	0.37±0.07^a^
C18:0	10.96±1.02^a^	15.66±1.73^b^	10.60±2.30^a^	9.11±1.98^a^	12.06±2.82^a,c^	12.72±1.78^b,c^
C20:0	0.05±0.01^a^	0.10±0.02^b^	0.07±0.01^a^	ND	ND	ND
C22:0	0.16±0.02^a^	0.38±0.11^b^	0.21±0.07^a^	0.07±0.01	0.21±0.15	0.18±0.05
C23:0	0.17±0.03^a^	0.25±0.04^b^	0.15±0.06^a^	ND	ND	ND
C24:0	0.39±0.04^a,b^	0.56±0.16^a^	0.32±0.15^b^	0.09±0.04	0.32±0.30	0.25±0.11
C16:1n-9	0.30±0.03	0.26±0.03^a^	0.38±0.10^b^	0.27±0.05	0.33±0.09	0.23±0.07
C16:1n-7	3.42±0.81^a^	0.75±0.31^b^	1.52±0.83^b^	4.00±1.66^a^	1.06±0.60^b^	1.15±0.63^b^
C18:1n-9	8.82±0.90^a^	7.91±1.76^a^	14.77±5.37^b^	10.85±3.15	10.83±3.66	7.11±2.49
C18:1n-7	5.62±0.99^a^	3.27±0.79^b^	3.36±1.02^b^	3.55±0.25^a,b^	4.33±1.06^a^	2.66±0.72^b^
C18:3n-3	0.39±0.10^a^	0.16±0.07^b^	0.22±0.06^b^	0.60±0.24^a^	0.20±0.11^b^	0.22±0.17^b^
C20:5n-3	0.27±0.05^a^	0.12±0.09^b,c^	0.20±0.11^a,c^	0.09±0.01^a^	0.02±0.01^b^	0.03±0.01^b^
C22:5n-3	1.98±0.22	1.93±0.19	2.10±0.47	2.20±0.35	2.62±0.55	2.67±0.21
C22:6n-3	6.63±0.93^a,b^	8.62±2.61^a^	4.93±1.48^b^	9.84±2.20	8.34±1.90	8.35±1.39
C18:2n-6	17.08±1.36	14.72±1.93	15.55±2.12	21.29±2.59	17.23±3.25^a^	14.91±4.02^b^
C18:3n-6	0.16±0.04^a^	0.47±0.18^a^	1.01±0.42^b^	ND	ND	ND
C20:3n-6	0.96±0.39	0.53±0.06	0.75±0.20	0.57±0.16	0.70±0.19	0.79±0.08
C20:4n-6	16.68±0.97^a^	18.78±1.10^a^	14.02±2.46^b^	9.30±1.82^a^	14.33±3.26^a,c^	18.85±4.27^b,c^
C22:4n-6	0.58±0.09^a^	1.28±0.42^b^	2.09±0.60^c^	0.28±0.07	0.47±0.15	0.51±0.25
C22:5n-6	0.25±0.09^a^	2.06±0.74^b^	2.50±1.02^b^	0.19±0.05^a^	0.67±0.17^b^	0.91±0.22^b^
ΣSFA	35.76±0.99	38.13±1.89	35.55±3.81	34.10±1.85^a^	35.10±2.24^a,b^	37.68±1.10^b^
ΣMUFA	18.79±1.79^a,b^	12.90±2.69^a^	20.76±6.70^b^	18.66±4.76^a^	16.54±5.18^a,b^	11.15±3.58^b^
Σn-3 PUFA	9.27±1.01^a,b^	10.83±2.64^a^	7.45±1.89^b^	12.73±2.26	11.17±2.33	11.28±1.35
Σn-6 PUFA	36.04±1.16	38.06±1.52	36.19±2.60	31.63±2.30^a^	33.39±0.46^a^	35.97±0.84^b^
C18:1n-9/C18:0	0.81±0.10^a,b^	0.52±0.15^a^	1.56±1.00^b^	1.30±0.60	0.98±0.50	0.58±0.25
C20:4n-6/C18:2n-6	0.98±0.10^a,b^	1.30±0.22^a^	0.93±0.29^b^	0.45±0.13^a^	0.89±0.39^a,b^	1.46±0.91^b^

Abbreviations: FA, fatty acid; ND; not determined.

Results are presented as means±s.d. Means between groups were tested with one-way ANOVA using Tukey *post hoc* test. Means in a row within the same organ with different letters are significantly different (*P*<0.05).

**Table 4:** Relative expression of genes in epididymal white adipose tissue and liver

	*Sham (*N=*7)*	*Duodenal switch (*N=*5)*	*Sleeve gastrectomy+duodenal switch (*N=*5)*
*White adipose tissue*			
Acaca	1.00±0.24^a^	0.49±0.04^b^	0.71±0.27^a,b^
Slc2a4	1.00±0.19^a^	0.42±0.03^b^	0.58±0.26^b^
LDLr	1.00±0.58^a^	0.27±0.23^b^	0.30±0.29^b^
			
*Liver*			
Acaca	1.00±0.15	1.15±0.39	1.36±0.64
HMG-CoA reductase	1.00±0.44^a^	2.67±1.15^a,b^	2.98±1.92^b^
Soat2	1.00±0.18^a^	1.74±0.16^b^	1.51 ±0.37^b^
CYP7A1	1.00±0.33	1.30±0.80	1.53±0.60
LDLr	1.00±0.16	0.88±0.26	0.80±0.13
Slc2a2	1.00±0.07^a^	0.71±0.18^b^	0.71±0.19^b^

Abbreviations: Acaca, acetyl-CoA carboxylase; CYP7A1, cholesterol 7 alpha-hydroxylase; LDLr, low-density lipoprotein receptor; Slc2a2, glucose transporter GLUT2; Slc2a4, glucose transporter GLUT4; Soat2, sterol *O*-acyltransferase.

Results are shown relative to 18S rRNA and normalized to Sham for each tissue, and are presented as means±s.d. Means between groups were tested with one-way ANOVA using Tukey *post hoc* test. Means in a row with different letters are significantly different (*P*<0.05).

**Table 5:** Plasma levels of cytokines

*pg* *ml*^*−1*^	*Sham (*N=*3)*	*Duodenal switch (*N=*5)*	*Sleeve gastrectomy+duodenal switch (*N=*5)*
IL-1a^1^	94±58^a,b^	31±21^a^	125±19^b^
IL-1b	310±303	29±18	215±196
IL-2	190±69^a,b^	85±55^a^	373±155^b^
IL-4	39±30^a,b^	6±5^a^	42±22^b^
IL-5^2^	129±45^a^	31±15^b^	106±42^a^
IL-6^3^	217±116	<LOD	158±110
IL-10	535±242	252±287	588±165
IL-12 p70	106±111	<LOD	75±77
IL-13^4^	61±58	<LOD	55±39
GM-CSF	41±43	<LOD	31±28
IFNγ^5^	77±66	19±9	75±54
TNFα^4^	66±80	<LOD	57±59

Abbreviations: GM-CSF, granulocyte-macrophage colony stimulating factor; IFNγ, interferon gamma; IL, interleukin; LOD, limit of detection; TNFα, tumour necrosis factor alpha.

Results are presented as means±s.d. Means between groups were tested with one-way ANOVA using Tukey *post hoc* test. Means in a row with different letters are significantly different (*P*<0.05).

1 *N*=3 in the DS group.

2 *N*=4 in the DS group.

3 *N*=2 in the sham group.

4 *N*=4 in the SG+DS group.

5 *N*=2 in the DS group.
